# Cost-Effectiveness of Pitavastatin in Dyslipidemia: A Systematic Review

**DOI:** 10.3390/healthcare14131847

**Published:** 2026-06-25

**Authors:** Nam Xuan Vo, Huong Lai Pham, Tan Trong Bui, Tien Thuy Bui

**Affiliations:** 1 Faculty of Pharmacy, Ton Duc Thang University, Ho Chi Minh City 700000, Vietnam; laihuong49@gmail.com; 2 School of Medicine, University of Medicine and Pharmacy, Ho Chi Minh City 700000, Vietnam; bttan.y22@ump.edu.vn; 3 Faculty of Pharmacy, Le Van Thinh Hospital, Ho Chi Minh City 700000, Vietnam; bttien.ths.tcqld23@ump.edu.vn

**Keywords:** pitavastatin, dyslipidemia, hypercholesterolemia, systematic review, cost-effectiveness, QALY

## Abstract

**Objectives**: Dyslipidemia is a major driver of cardiovascular disease (CVD), causing a global economic burden. Statins are the mainstay for reducing LDL-C, with pitavastatin (PIT) being the newest-generation statin, showing non-inferior efficacy compared with potent statins. This study aims to assess the cost-effectiveness of pitavastatin in comparison with atorvastatin (ATOR) and rosuvastatin (ROS). **Method**: The PubMed, Cochrane, and Embase databases were searched to identify full or partial economic evaluations through 19 November 2025. Our primary outcome is the incremental cost-effectiveness ratio (ICER), with health outcomes measured by quality-adjusted life years (QALYs) or percentage reduction in LDL-C. Regarding quality assessment, the Consolidated Health Economic Evaluation Reporting Standards (CHEERS) 2022 tool was applied. The Revised Cochrane Risk of Bias Tool for Randomized Trials (RoB 2) checklist and Risk Of Bias In Non-randomized Studies of Interventions (ROBINS-I) were performed for RCTs and non-RCT risk assessments, respectively. **Result**: Five studies were synthesized. One model-based analysis over a lifetime revealed that PIT was less expensive but generated slightly fewer QALYs than ATOR and was dominated by ROS. Four within-trial CEAs with follow-up ≤12 months found that for each 1% reduction in LDL-C, PIT was generally more economical than low-dose ATOR but consistently more costly than ROS. **Conclusions**: Because of the small number and heterogeneity of studies, it is not possible to draw firm conclusions about the cost-effectiveness of PIT. Further model-based analyses with an adequate sample size and comprehensive costing are needed to clarify the economic role of PIT.

## 1. Introduction

Dyslipidemia is a condition in which there is an abnormality in at least one of the blood lipid parameters, including total cholesterol (TC), triglyceride (TG), low-density lipoprotein cholesterol (LDL-C), and high-density lipoprotein cholesterol (HDL-C) [1,2]. The most common condition is high LDL-C combined with normal or low HDL-C, with elevated LDL-C being a key modifiable risk factor for atherosclerosis and cardiovascular disease (CVD) [3,4]. In 2019, approximately 44% of deaths due to ischemic heart disease and 22% of deaths due to ischemic stroke were attributed to excessive LDL-C [5]. A 2017 study found that increased LDL-C was responsible for 19.71% of cardiovascular deaths [6]. In Austria in 2019, 8.2% of all deaths were attributed to hypercholesterolemia [2]. Especially in the elderly aged 60–89, elevated LDL-C levels are associated with a threefold higher risk of death compared to younger individuals [7]. More concerningly, the rising incidence of hyperlipidemia in recent reports has directly increased the risk of CVD and consequently aggravated the financial burden on healthcare systems [4,8]. For example, a meta-analysis estimated the prevalence of dyslipidemia in China to be 42% [6], while in Korea, the number of diagnosed cases increased sharply from 1.5 million in 2002 to 11.6 million in 2018 [9]. In addition, 75% of people with dyslipidemia also have other underlying diseases, such as hypertension or diabetes, making treatment even more complicated [9]. Overall, dyslipidemia is not only a global issue due to its high prevalence but also because of its potential to cause complications and comorbidities, making disease management more difficult.

The economic burden of dyslipidemia is enormous. According to 2008 US statistics, dyslipidemia is among the 10 most expensive medical conditions to treat [3,10]. Medical costs for dyslipidemia are expected to increase by 22% from 2020 to 2050, from 54 billion USD to 66 billion USD [11]. Of these, direct costs are considered the primary reason for the increase in total costs associated with treating dyslipidemia. In Austria, the total number of cases of hypercholesterolemia was 806 million EUR, of which nearly 40% were direct medical costs [2]. In Mexico, inpatient treatment costs accounted for 75–89% of the total costs of treating dyslipidemia in patients at high risk of CVD [12].

Statins are the first-line treatment for dyslipidemia. Statins are an effective therapy to reduce LDL-C concentration to optimal levels, thereby lowering the risk of ASCVD [8,13,14,15,16]. High-intensity statins are effective, safe, and capable of reducing LDL-C by 50% [17]. In European countries, lipid-lowering drugs, mainly statins, are the second most commonly consumed pharmacological group after antihypertensives, highlighting both the importance of this class in healthcare and the need to evaluate its economic benefits to optimize system costs [18]. Among them, pitavastatin—the latest generation of approved statins—has attracted the attention of medical experts thanks to its effectiveness and safety, which are not inferior to those of statins widely used on the market, such as atorvastatin and rosuvastatin. In developed countries such as China and Japan, real-world data indicate that pitavastatin is prescribed frequently in routine care alongside rosuvastatin and atorvastatin, although with a smaller prescription share [19,20]. Previous cost-effectiveness analyses, such as a Markov model in Taiwan that simulated 10,000 patients over ten years, have shown that initiating statin therapy (simvastatin, rosuvastatin, atorvastatin, or pitavastatin) can prevent cardiovascular events and yield substantial gains in both costs and QALYs [21]. Yet, the economic benefits of pitavastatin have only been examined superficially within the statin class as a whole, and no study has specifically focused on the cost-effectiveness of pitavastatin monotherapy compared with other statin options. Therefore, this study will provide an overview of the cost-effectiveness of pitavastatin in comparison with atorvastatin and rosuvastatin, from the past to the present.

## 2. Method

### 2.1. Searching Strategy

The study was conducted in January 2025, with the final review on 19 November 2025. The data presentation was based on the Preferred Reporting Items for Systematic Reviews and Meta-Analyses (PRISMA) guidelines [22]. Studies for the economic analysis were sourced from three major high-impact databases, including PubMed, Cochrane, and Embase, with no time restriction applied. English keywords related to the topic were entered into the search bar and combined using the Boolean algorithm: “AND”, “OR”, or “NOT” to construct full search strings. Multiple search strategies were employed to ensure that all studies within the research scope were captured, as outlined in Supplemental Table S1. The full syntax covering all included studies was set up as follows: (pitavastatin) AND ((cost) OR (cost-effective) OR (cost-effectiveness)). Moreover, the review has been registered in the Open Science Framework using the following link: https://osf.io/mxkjf accessed on 2 July 2025.

### 2.2. Selection Process and Criteria for Research Selection

The PICO framework was initially applied to define the main scope of the research:

(1) P—Participant: patients diagnosed with dyslipidemia or those who had a baseline of LDL-C > 100 mg/dL.
(2) Intervention: pitavastatin as the sole treatment.
(3) C—Comparison: rosuvastatin or atorvastatin as monotherapy.
(4) Outcome: ICERs, cost, and effectiveness (QALY, LDL-C reduction, etc.).

Based on this framework, more specific inclusion criteria were established to ensure clarity in identifying suitable studies. A study was considered eligible if it met the following conditions. (1) Research designs that fell within the economic domain were prioritized, such as cost-effectiveness analysis (CEA), cost-minimization analysis (CMA), cost–benefit analysis (CBA), cost-utility analysis (CUA), or economic evaluation. In addition, Randomized Controlled Trials (RCTs) that specified a cost assessment were also accepted. (2) The subject of the study was humans. (3) The study mentioned pitavastatin monotherapy. (4) The analysis focused on dyslipidemia, characterized by an abnormal increase in LDL-C values. (5) The primary outcome included ICERs. (6) The whole text was accessible and presented in English.

In contrast, studies were excluded if they violated any of the following conditions: (1) the study design was not in the field of health economics, or (2) the clinical trial did not include a cost assessment section. (2) The subject of the study was animals. (3) The study focused on pitavastatin in combination. (4) The analysis did not focus on dyslipidemia, or the patients had normal LDL-C levels at the beginning, or hyperlipidemia in HIV patients. (5) The main results did not mention the ICERs or failed to provide sufficient information to determine whether pitavastatin was cost-saving. (6) Full-text access was not retrievable, or the publication was not in English.

Based on the database results, the studies were screened by comparing each article manually against the predefined inclusion and exclusion criteria. Titles and abstracts were reviewed first. Then, the full texts were assessed to verify the validity of key data (such as cost, ICERs, etc.), and relevant information was extracted. Two independent researchers conducted the entire process, from preliminary screening to data synthesis. Notably, during the screening process, the researchers cross-checked each other’s results after generating the list of eligible full-text articles. Any disputes or disagreements were resolved by discussion until both researchers reached an agreement.

### 2.3. Data Extraction

After accessing the full-text, the following basic information was extracted manually: author name, year of publication, study design, country, intervention and comparison group, indication, mean baseline lipid profile, age of participants, number of patients, model types, acquired perspective, discount rate, time horizon, duration of study, setting, and kind of sensitivity analysis performed.

The primary outcome is the incremental cost-effectiveness ratio (ICER). ICERs are indices used in health economics analysis to compare the costs and effectiveness of a new intervention or treatment with existing methods. This index helps assess whether an intervention is worth investing in based on the cost per unit of effectiveness, usually measured in quality-adjusted life year (QALY) [23]. This study employs two types of costs: direct costs and drug costs. Direct costs include all expenses directly related to health, such as nursing care and medical treatment costs. Additionally, the results of deterministic sensitivity analysis (DSA) and probabilistic sensitivity analysis (PSA) were collected to assess the uncertainty associated with ICERs.

Similarly, all of the data collection was performed by two independent researchers. Although the fundamental information and the primary outcome were extracted in parallel, we prioritized confirming the primary outcome data, such as cost/QALY or cost/%LDL-C reduction. Then the researchers cross-checked and finalized the general study information.

### 2.4. Data Synthesis

In this phase, key data from each article were manually extracted, analyzed, compared, and summarized. Due to the heterogeneity of economic outcomes across studies, a narrative synthesis was adopted and conducted in accordance with the 9-item SWiM guideline [24]. Regarding baseline information, LDL-C concentrations and statin doses were noted to estimate the severity of dyslipidemia in each study. Diagnoses were also considered to enhance the validity of comparisons. Additionally, the countries where the studies were conducted were recorded to observe prescription trends for dyslipidemia treatments in the United States and Asia. A summary table was used to present this information, with variables expressed as mean ± standard deviation (SD) or ± standard error of the mean (SE).

On the other hand, costs and effectiveness were prioritized for extraction and comparison to assess the primary outcome. The types and total costs over specific periods were documented to determine which components contributed most to the overall cost of pitavastatin relative to other interventions. The primary outcome—the cost-effectiveness of pitavastatin—was evaluated based on the types of health outcomes reported. When effectiveness was expressed as a percentage reduction in LDL-C, cost-effectiveness was presented as the cost per percentage point reduction in LDL-C. When effectiveness was measured in QALYs, cost-effectiveness was assessed using the cost per QALY. The ICERs in each analysis were then compared to the respective country’s willingness-to-pay (WTP) threshold to determine whether pitavastatin was considered cost-effective. Finally, results across the studies were compared to identify trends in the cost–benefit profile of pitavastatin.

In addition, where possible, results from DSA and PSA were used to confirm the robustness of ICERs. DSA identified which parameters had the most decisive influence on ICER outcomes. At the same time, PSA estimated the likelihood that pitavastatin would be considered cost-effective under each country’s WTP threshold. Together, these analyses strengthened the conclusion regarding the cost-effectiveness of pitavastatin across studies.

### 2.5. Quality Assessment

The Consolidated Health Economic Evaluation Reporting Standards 2022 (CHEERS 2022) tool was used to assess the quality of eligible economic analyses [25]. This is a 28-criterion assessment that focuses on essential information in an economic analysis. Each study will be evaluated, converted into a score based on the amount of information provided, and the quality of its conclusions will be determined. There are three scoring options: “1” for complete details provided, “0.5” if the analysis only meets half of the requirements or contains ambiguous information, and “0” if no information is relevant to the question. With a total score of 28, analyses that score 22 or above are determined as “good” quality. A total score ranging from 14 to 21.5 is considered “moderate” quality, and articles below 14 points are considered “poor”-quality research. Additionally, clinical trials were not assessed for quality because they were outside the economic scope, and the data extracted for the cost assessment section were limited.

### 2.6. Risk of Bias

The included trial-based analysis underwent the risk of bias assessment using the Revised Cochrane Risk of Bias Tool for Randomized Trials (RoB 2) checklist for randomized study design [26], and Risk Of Bias In Non-randomized Studies of Interventions (ROBINS-I) for non-randomized studies [27]. Based on five domains [26] and seven domains [27], respectively, both tools aim to assess potential bias in study design, methodology in the selected patients, and the intervention. Following the tool algorithm, each domain judgment can be rated as Low, Moderate, Serious, or Critical level, indicating an increasing level of risk of bias and a corresponding decrease in study quality. The final judgment of the studies is determined by the worst risk of bias within domains.

## 3. Results

### 3.1. Selection Process

**Figure 1:**
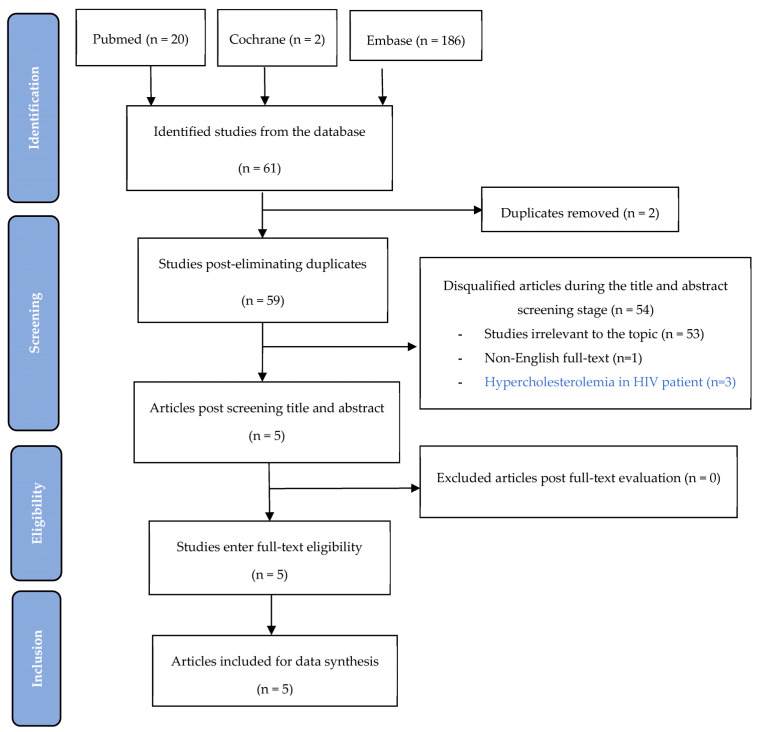
PRISMA flow diagram for identifying and selecting articles.

The searching and screening process for article selection, following the PRISMA guideline, is shown in Figure 1. A total of 61 studies were identified, comprising 20 from PubMed, two from Cochrane, and 186 from Embase. Notably, the review was conducted three times, and the last search was retrieved on 11 November 2025. Following the removal of two duplicates, the total number of studies dropped to 59. Each article was initially evaluated for the relevance of its title and the sufficiency of its abstract, resulting in five studies being excluded for failing to meet the inclusion criteria. The remaining five works underwent full-text evaluation according to the defined exclusion criteria. Hand searching on Google Scholar was also performed, yielding no results because the query was not conducted in English. Ultimately, all five reports satisfied the eligibility requirements and proceeded to the data synthesis step.

### 3.2. Characteristics of Selected Articles

The general characteristics of the eligible trials and cost analyses are summarized in Table 1. A total of five studies analyzed the economic aspects of statins. Among them, four out of five publications were within-trial evaluations, with three trials originating from RCTs [28,29,30] and the remaining one being an observational study [31]. Only one study is designed as a cost-utility analysis. As a result, information regarding model type, discount rate, analytical perspective, and the choice of sensitivity analysis was reported only in this study [32], while these elements are not reported in the other four studies [28,29,30,31]. The time horizon was recorded as lifetime in the CUA [32], whereas in the trial-based analyses, the time horizon tended to align with the treatment duration, ranging from 8 weeks to less than 12 months [28,29,30,31]. All five studies were conducted in Asian settings, including Korea [31], Japan [28], Thailand [29], India [30], and Vietnam [32]. Participants were adults, with sample sizes ranging from 98 to over 5500.

Additionally, differences were observed in the methods used to compare the interventions. Four out of five studies (80%) directly compared statin monotherapy regimens. The remaining study evaluated statin groups based on their ability to reduce the percentage of LDL-C, in which the high-dose group (atorvastatin 40 mg, rosuvastatin 20 mg) was compared with the medium-high-dose group (atorvastatin 20 mg, rosuvastatin 10 mg), the medium-low-dose group, the low-dose group, and the group using the combination of simvastatin and ezetimibe [31]. In terms of indications, most studies (three out of five) used statins to treat dyslipidemia in patients who had an initial LDL-C level above 100 mg/dL [28,29,31,32]. The other two studies were conducted in comorbid populations, including those with chronic kidney disease [28] and type 2 diabetes [30].

### 3.3. Quality Assessment

The CHEERS 2022 quality evaluation of the included studies is summarized in Table 2. The overall quality of the included evaluations ranged from moderate to good. Among them, only one full economic evaluation was deemed “good” quality, scoring 25 points [32].

In contrast, the four trial-based cost-effectiveness analyses did not report several critical economic components, including item 4, “Framework for conducting the economic analysis”; item 8, “Perspective”; item 10, “Discount rate”; and item 16, “Model selection and rationale”. Additionally, these trial-based cost analyses lacked formal assessment of economic uncertainty or scenario analysis, resulting in very low scores (ranging from 0 to 0.5) for items 20, “Characterizing uncertainty,” and 24, “Assessing the effect of uncertainty.” Consequently, the four within-trial CEAs received total scores ranging from 15.5 to 16.5 points, resulting in a classification of “moderate” quality [28,29,30,31]. Overall, all five included reports failed to meet the requirements for item 19, “Analyzing how effects vary across populations,” and item 25, “Engagement level of study participants.”

### 3.4. RoB 2 and ROBIN-I

The risk of bias resulting from randomized trials is illustrated in Table S2. Only one out of the three studies was assessed as having a Low risk of bias [28]. The other two studies did not provide sufficient information to confirm that the randomization sequence was concealed, resulting in Domain 1 being rated as having “Some concerns.”[29,30]. The study by Devi et al. [30] showed the lowest quality as it excluded a significant number of participants post-randomization, with no statistical method to estimate the impact of missing data. As a result, the final judgment was recorded as High risk of bias [30].

Similarly, the assessment of the methodology of one included non-RCT is demonstrated in Table S3. The study did not control for confounding factors such as BMI, sex, and comorbidities, demonstrating a potential bias in estimating LDL-C reduction effectiveness [31]. The study did not include patients who discontinued the drug or had changes in statin dose in the primary outcome analysis, thereby creating an artificially ‘clean’ population profile (Domain 3, 4, 5). Consequently, the study was scored as a Serious risk of bias [31].

### 3.5. Cost-Effectiveness-Related Data

#### 3.5.1. %LDL-C Reduction as the Measure of Effectiveness

Details comparing the costs and effectiveness of pitavastatin with other statins are illustrated in Table 3. A 2/2 analysis found that pitavastatin was costlier than rosuvastatin, leading to a higher overall price per %LDL-C reduction [28,31]. For a 10 mg/dL reduction in LDL-C, the option with rosuvastatin in a year saves $ 28.57 compared to pitavastatin [28]. Notably, reducing LDL-C by 1% with PIT would cost twice as much as with ROS annually (8.0 USD vs. 4.5 USD), suggesting that PIT is less cost-effective than ROS in Japan and Korea.

On the other hand, pitavastatin was more cost-effective than atorvastatin in 2/3 analyses, as the PIT drug expense is cheaper than ATOR [29,31]. In Thailand, the monthly drug cost per 1% LDL-C reduction for PIT is half the price compared to ATOR, at 0.77 USD and 1.56 USD, respectively [29]. This characteristic was also observed in a longer assessment period, such as Korea over 12 months, where the annual acquisition cost of PIT 1 ng was 33 USD lower than ATOR 10 mg (183 USD vs. 216 USD) [31]. However, the highest dose of PIT 4 mg was deemed less cost-beneficial than ATOR 20 mg, as it was inferior in terms of LDL-C improvement efficacy [30]. Collectively, based on the percentage LDL-C reduction, using pitavastatin 1–2 mg is cost-effective compared to atorvastatin, but more expensive than rosuvastatin.

#### 3.5.2. QALY as Effectiveness

A comparison of total cost, health outcomes, and ICER between pitavastatin and other statins is summarized in Table 4. In the LMIC setting, pitavastatin is more cost-effective than atorvastatin within Vietnam’s budget, as it incurs lower costs but yields fewer QALYs. When compared with rosuvastatin, pitavastatin required a higher investment but did not improve quality of life; it is an ineffective option [32]. Findings from DSA revealed that pitavastatin cost and utility were the most driving factors to ICERs [32].

## 4. Discussion

### 4.1. Cost per %LDL-C Reduction

Findings from trial-based CEAs consistently indicate that low-dose pitavastatin (1–2 mg) is generally less costly than atorvastatin but more expensive than rosuvastatin for achieving the same LDL-C lowering. This pattern was observed only in the short term (up to 8–12 weeks or 12 months), reflecting the acquisition cost of study doses rather than long-term cardiovascular outcomes. This is because the low dose of pitavastatin is comparable to atorvastatin in its LDL-C-lowering effect, making drug expense the most impactful factor for cost-effectiveness between the two interventions. But at high doses, the magnitude of LDL-C lowering appears to be a key driver of pitavastatin’s cost-effectiveness in the Indian context. As demonstrated by Devi et al., although pitavastatin 4 mg had a slightly lower total drug cost than atorvastatin 20 mg, it produced a smaller LDL-C reduction (25.0% vs. 29.1%), leading to a higher cost per 1% LDL-C reduction and making pitavastatin less cost-effective than atorvastatin [30].

Previous economic analyses have noted that statins are cost-effective only in patients at high risk of CVD, but not cost-saving in the low-risk group [33,34]. But in our included studies, although pitavastatin was not more favorable than atorvastatin in patients with type 2 diabetes in the Indian study, the same trial showed that pitavastatin provided more cost-effective improvements in fasting glucose, HbA1c, triglycerides, and HDL-C, suggesting that it may be a reasonable option in patients where glycemic control is prioritized alongside LDL-C reduction [30]. Supporting this, a recent pharmacovigilance analysis from 2025 reported that atorvastatin showed the strongest disproportionality signal for diabetes-related adverse events among licensed statins, while pitavastatin ranked third [35]. Although it is undeniable that atorvastatin has the highest prescription rate [36,37], it also causes adverse effects across multiple organ systems. Atorvastatin has been observed to have statin-associated neurological effects and is more hepatotoxic than rosuvastatin and pitavastatin [38,39]. Following a comprehensive AE analysis of more than 43,000 statin-treated cases in the USA, it was associated with the highest risk of metabolic disorders out of the seven licensed statins [40]. These may be helpful when choosing pitavastatin as a targeted alternative in patients with a high baseline diabetes risk. But since subgroup analyses were not performed, our evidence is largely restricted to general dyslipidemia. Thus, our review can only suggest a possible pattern: that pitavastatin might be more beneficial in certain high-risk patients, rather than establishing this as a generalized insight.

### 4.2. Cost per QALY

When effectiveness is measured in QALYs over a lifetime horizon, the picture is broadly similar. In the Vietnamese context, pitavastatin 2–4 mg was more cost-effective than atorvastatin 10–20 mg, yielding cost savings at the expense of a small QALY loss, resulting in an ICER below the national willingness-to-pay threshold. In contrast, pitavastatin was dominated by rosuvastatin; that is, pitavastatin incurred higher costs and yielded fewer QALYs than rosuvastatin. However, this QALY-based comparison of pitavastatin versus the two statins is based on a single full economic evaluation, which limits the generalizability of this finding.

Even so, model-based analyses from other countries have reached similar conclusions and provide a coherent explanation for why pitavastatin frequently fails to outperform rosuvastatin: at currently observed prices, pitavastatin often has a substantially higher acquisition cost than rosuvastatin [41,42]. In Spain, for example, the acquisition cost of pitavastatin was at least six times higher than that of rosuvastatin (0.24–0.48 €/mg vs. 0.04–0.09 €/mg), while rosuvastatin also produced greater LDL-C reductions and therefore fewer cardiovascular events over time. As a result, pitavastatin incurred higher total direct costs and generated lower QALYs and was thus dominated by rosuvastatin [41]. Similar findings were reported by Ikeda et al. in Japan, where pitavastatin 2–4 mg was consistently more expensive and less effective than rosuvastatin across both moderate and severe hypercholesterolemia scenarios [42]. In the Vietnamese setting of Vo et al.’s study, even though the downstream costs of cardiovascular events were not incorporated, the drug acquisition cost of pitavastatin 2 mg was estimated to be around 1.5 times higher than that of rosuvastatin 5 mg, which already resulted in pitavastatin being a dominated option [32]. Taken together, the existing CEAs suggest that, in the settings and time periods studied, the high drug cost of pitavastatin is a major barrier to its cost-effectiveness relative to rosuvastatin. As ROS has the most vigorous LDL-C reduction activity of all statins [4,43], it is expected that PIT’s additional cost is not offset by gains in QALYs, so pitavastatin typically appears as a dominated strategy in lifetime models. The DSA results also confirmed that the drug price is the factor with the most significant effect on the cost-effectiveness of pitavastatin [32]. Nevertheless, these interpretations should be treated with caution, given the small number of full economic evaluations and the strong context dependence of statin prices. This interpretation is also consistent with our findings from trial-based CEAs: pitavastatin had a higher drug cost than rosuvastatin to achieve the same short-term 1% LDL-C reduction. In Korea, for example, the cost per 1% LDL-C reduction with pitavastatin was approximately twice that of rosuvastatin [31].

### 4.3. Implications, Strengths and Limitations

It is important to note that the benefits of pitavastatin over atorvastatin are derived mainly from trial-based CEAs. Because these evaluations originated from RCTs and a real-world analysis and were conducted strictly within short follow-up periods, several critical economic elements were not reported. For example, the analytical perspective was unspecified, making it difficult to determine the full range of costs included and limiting the interpretation to drug acquisition costs within each country. The absence of sensitivity or scenario analyses also prevents assessment of whether the cost-effectiveness ranking would change if drug prices fluctuated by ±20% or if different dosing strategies were applied. Therefore, the positive economic evidence for pitavastatin cannot support its use as a cost-effective alternative to atorvastatin.

Several challenges limit pitavastatin’s popularity in the statin market. The biggest obstacles are low availability in healthcare settings and low affordability, especially in African and Southeast Asian countries [44]. According to statistics, in low- and middle-income countries (LMICs), the availability of statins in public healthcare facilities is 5.4% for generic drugs and almost none for branded drugs [44]. In private facilities, drug availability is higher, with rates of 13.3% and 35.9% for generic and branded medicines, respectively [44]. Statin prices also vary widely, from 1 to 62 USD [44]. Differences in drug policies across countries, especially in low- and middle-income countries (LMICs), also contribute to the limited introduction of pitavastatin into the market. In LMICs, government funding for medications is often restricted, so policymakers tend to prioritize drugs that treat acute conditions or provide short-term benefits. In dyslipidemia, most patients with elevated LDL-C are asymptomatic [45]. Since the treatment does not provide immediate effects, patients may not recognize the importance of taking the medication, leading to poor adherence and increased long-term costs of pitavastatin. The long-term effectiveness of statin treatment depends mainly on individual adherence and tolerability, rather than on CVD risk [46].

The general statin strategy in Western populations, particularly within Europe, is driven by clinical practice guidelines from the European Society of Cardiology (ESC), which are regularly updated to manage cardiovascular disease (CVD), the region’s leading cause of death. The 2016 guidelines, which establish specific targets for modifiable risk factors like smoking, blood pressure, and cholesterol, establish specific LDL-cholesterol (LDL-C) targets, primarily <1.8 mmol/L (70 mg/dL) for patients with established coronary heart disease, or a reduction of at least 50% for those with baseline levels between 1.8 and 3.5 mmol/L [47]. Standardized intensification protocols for reaching these goals involve initiating Simvastatin at various doses, titrating upward, switching to stronger statins such as atorvastatin, and finally adding ezetimibe for resistant cases [47]. From a cost and cost-effectiveness perspective, the study calculated a base-case incremental cost-effectiveness ratio (ICER) of 52,968 € per quality-adjusted life year (QALY), noting that the strategy becomes significantly more efficient when focusing on elderly patients, those with high baseline risks, or when using less stringent targets [47]. However, the risks and hazards remain significant, as patients face a high 10-year probability of recurrent vascular events, such as non-fatal myocardial infarction and stroke, which is further exacerbated by real-world challenges like inadequate medication compliance and insufficient risk factor control [47].

This is the first comprehensive study to date that analyzes the cost-effectiveness of pitavastatin. Direct costs were primarily considered in the study to be a strength as they have the greatest influence on the total cost of dyslipidemia treatment. Aside from determining the economic benefits of pitavastatin compared to other statin groups, the study also highlighted obstacles in current conditions and policies that hinder the implementation of pitavastatin on a larger scale. At the same time, the study mentioned the economic benefits of PIT and the two most prominent statin groups in clinical practice. However, the limited number and types of pharmacoeconomic study designs mean that most available CEAs focus on clinical benefits and drug costs over short periods and do not fully capture the broader spectrum of costs faced by patients with hypercholesterolemia and multiple comorbidities, so the subgroup analysis and healthcare setting were not applied in this research. With the cost-effectiveness of pitavastatin determined by two independent outcomes, our study findings were classified as surrogate-based economic evidence and long-term health outcome evidence. Such methodological differences may restrain direct comparison between the studies and thus weaken the strength of the overall conclusion. Focusing on direct costs, the included trial-based CEAs primarily considered acquisition costs during relatively short observation periods of 1–12 months. Although adverse event management and hospitalization costs may have been limited during the initial treatment period, these studies may still overlook relevant medical costs such as lipid panel testing and liver function monitoring. Consequently, this makes the cost-analytical perspective relatively narrow, failing to reflect comprehensive cost-effectiveness in terms of cost per % LDL reduction.

Another major limitation of our findings is their limited generalizability to Western populations, as all included trials were conducted in Asia. This geographical bias is significant due to the substantially lower willingness-to-pay (WTP) thresholds in Asian countries, such as Thailand and the Philippines, compared to Western nations like the U.S., Canada, and Germany [48,49]. According to Riviere et al.’s 2023 report, the average health expenditure per capita (HEpc) in the U.S. is estimated at 10,921 USD per year, which is 20 to 280 times higher than that of some Asian countries [48]. Such vast disparities in healthcare budgets and economic priorities reinforce that cost-effectiveness findings are highly context-specific and may not remain valid in different economic settings. In addition, with a relatively small number of studies, sample sizes vary widely, from around 100 participants in RCTs to more than 5000 in observational analyses; although this combination provides both internal and external validity, it also introduces considerable heterogeneity. Consequently, this review is unable to establish definitive conclusions regarding the cost-effectiveness of pitavastatin and is better viewed as describing a collective pattern across study contexts.

## 5. Conclusions

In conclusion, the cost-effectiveness of pitavastatin cannot be determined due to the limited number of available studies, heterogeneity in study designs, and methodological limitations in the included economic evaluations. Existing trial-based analyses suggest that low-dose pitavastatin may be more cost-effective than atorvastatin for achieving a 1% reduction in LDL-C over the short term. Over a lifetime horizon, the only available model-based evaluation indicates that pitavastatin is more economical but yields slightly fewer QALYs than atorvastatin. Across both types of effectiveness outcomes, short-term LDL-C reduction and lifetime QALYs, pitavastatin is consistently dominated by rosuvastatin. Further full cost-effectiveness studies using economic models that incorporate downstream cardiovascular event costs and subgroup analyses by comorbidity are needed to clarify the specific economic role of pitavastatin and to allow more definitive conclusions.

## 6. Future Directions

Future research on pitavastatin in dyslipidemia should move beyond short-term, trial-based cost comparisons toward robust, long-term model-based evaluations that better capture clinically meaningful outcomes, such as reductions in cardiovascular events and lifetime quality-adjusted life years (QALYs). The current evidence remains limited by small sample sizes, short time horizons (≤12 months), and substantial methodological heterogeneity, which restricts the generalizability of findings and precludes definitive conclusions regarding its economic value relative to atorvastatin and rosuvastatin. Notably, the absence of comprehensive modeling frameworks, discounting strategies, and uncertainty analyses in most of the included studies underscores the need for standardized economic evaluation designs aligned with the CHEERS 2022 recommendations. Future studies should incorporate Markov or microsimulation models with longer time horizons, societal or healthcare payer perspectives, and sensitivity analyses to better reflect real-world decision-making contexts, particularly in low- and middle-income countries where cost constraints are critical.

In addition, emerging evidence suggests that pitavastatin may offer distinct advantages in specific patient subgroups, particularly those with metabolic comorbidities such as type 2 diabetes, where its relatively favorable glycemic and safety profile could translate into long-term economic and clinical benefits. However, this hypothesis remains underexplored due to a lack of subgroup-specific analyses and real-world effectiveness data. Future directions should therefore prioritize stratified economic evaluations integrating patient heterogeneity, pharmacovigilance data, and real-world evidence to identify populations in which pitavastatin may provide optimal value. Furthermore, head-to-head comparative effectiveness research that incorporates both lipid and non-lipid outcomes, as well as indirect costs and adherence-related factors, will be essential to fully elucidate the role of pitavastatin within contemporary lipid management strategies.

## Figures and Tables

**Table 1:** Overall features of included studies.

No	Author, Country, Year	Study Design	Interventions	Indication	N	Mean Baseline (±SD)	Age	Discount Rate	Time Horizon	Model	Perspective	Conduct Place	SA
1	Abe et al., Japan 2015 [2,28]	RCT	PIT 1–2 mg vs. ROS 2.5 mg	Dyslipidemia (LDL-C ≥ 100 mg/dL in CKD patients	134	LDL: ≥130 TC: ≥220 150< TG < 200 HDL-C: ≥50	20–80	-	12 months	-	-	Nihon University School of Medicine	-
2	Sansanayudh et al., Thailand 2010 [3,29]	RCT	PIT 1 mg vs. ATOR 10 mg	Dyslipidemia	98	TC: ≥220 LDL-C: ≥130 TG: ≤150	≥18	-	8 weeks	-	-	Phramongkutklao Hospital, Bangkok	-
3	Devi et al., India 2025 [6,30]	RCT	PIT 4 mg vs. ATOR 20 mg	ASCVD prevention in T2DM patients	84	LDL: ≥ 130 TC: ≥ 215 150 < TG < 200 HDL-C ≤ 50	35–70	-	12 weeks	-	-	Medicine outpatient department (OPD) of Guru Nanak Dev Hospital of Government Medical College	-
4	Jeong et al., Korea 2017 [1,31]	CEA	High-dose Statin ^s1^ vs. high-to mod-dose Statin ^s2^ vs. mod-low dose Statin ^s3^ vs. low-dose Statin ^s4^ vs. SIM + EZE	Dyslipidemia (LDL-C ≥ 100 mg/dL)	5579	LDL-C: 147 ± 30 ^S.D^ TC: 226 ± 39 ^S.D^ TG: 166 ± 104 ^S.D^ HDL-C: 50 ± 13 ^S.D^	≥18	-	12 months	-	-	Seoul St. Mary’s Hospital	-
5	Vo et al., Vietnam 2025 [7,32]	CUA	PIT 2–4 mg vs. ATOR 10–20 mg vs. ROS 5–10 mg	Dyslipidemia	5579	LDL-C: 147 ± 30 ^S.D^ TC: 226 ± 39 ^S.D^ TG: 166 ± 104 ^S.D^ HDL-C: 50 ± 13 ^S.D^	≥18	3	14 years	Markov	Healthcare	South Korea	DSA

Abbreviation: ASCVD: atherosclerotic cardiovascular disease; CEA: cost-effectiveness analysis; CUA: cost-utility analysis; CKD: chronic kidney disease; T2DM: type 2 diabetes mellitus; HDL-C: high-density lipoprotein cholesterol; LDL-C: low-density lipoprotein cholesterol; TC: total cholesterol; TG: triglyceride; PIT: pitavastatin; SIM: simvastatin; EZE: ezetimibe; DSA: deterministic sensitivity analysis; RCT: Randomized Controlled Trial; ^S.D^: standard deviation. ^s1^: atorvastatin 40 mg, rosuvastatin 20 mg. ^s2^: atorvastatin 20 mg, rosuvastatin 10 mg. ^s3^: atorvastatin 10 mg, pitavastatin 2 mg, pravastatin 40 mg, rosuvastatin 5 mg, simvastatin 20 mg. ^s4^: pravastatin 20 mg, pravastatin 10 mg.

**Table 2:** Quality of economic analysis following CONSORT 2022 criteria.

No.	Items	Abe et al., Japan 2015 [28]	Sansanayudh et al., Thailand 2010 [29]	Devi et al., India 2025 [30]	Jeong et al., Korea 2017 [31]	Vo et al., Vietnam 2025 [32]
1	Title	0.5	0.5	0.5	1	1
2	Abstract	1	1	1	1	1
3	Context and research aims	1	1	1	1	1
4	Framework for conducting economic analysis	0	0	0	1	1
5	Participants	1	1	1	1	1
6	Settings and conducting place	1	1	1	1	1
7	Interventions and comparators	1	1	1	1	1
8	Perspective	0	0	0	0	1
9	Time horizon	1	0.5	0.5	0.5	1
10	Discount rate	0	0	0	0	1
11	Outcomes determination	1	1	1	1	1
12	Tool to measure outcome	1	1	1	1	1
13	Outcome valuation	0.5	0.5	0.5	0.5	1
14	Evaluation of healthcare inputs and expenditures	1	1	1	1	1
15	Currency conversion	1	1	1	1	1
16	Model selection and rationale	0	0	0	0	1
17	Assumptions	0.5	0.5	0.5	1	1
18	Heterogeneity identification	0	0	0	1	0
19	Analyzing how effects vary across populations	0	0	0	0	0
20	Uncertainty identification	0	0	0	0.5	1
21	Engagement plan for affected populations	0	0	0	0	1
22	Inputs and parameters within the study	1	0.5	1	1	1
23	Summary of the main result	1	1	1	1	1
24	Determining the level of uncertainty	0	0	0	0.5	1
25	Engagement level of study participants	0	0	0	0	0
26	Main findings, limitations, overall, current point of view	1	1	1	1	1
27	Sponsor mention	1	1	1	1	1
28	Conflict of interest	1	1	1	1	1
	Total score	16.5	15.5	16	20	25
	Conclusion	Moderate	Moderate	Moderate	Moderate	Good

**Table 3:** Comparison of drug costs and effectiveness between pitavastatin and other statins from trial-based evaluation.

Country, Currency	Comparison	12-Month Drug Cost	Effectiveness	Cost per % LDL-C Reduction	Conclusion
**Pitvastatin vs. Rosuvastatin**					
Japan, 2012 USD [28]	PIT 1–2 mg vs. ROS 2.5 mg	82.23 vs. 53.66	10 mg/dL LDL-C reduction	-	PIT is not as cost-effective as ROS in CKD patients
Korea, 2016 USD [31]	PIT 2 mg vs. ROS 5 mg	183.1 vs. 112.9	% LDL-C reduction	8.0± 0.6 ** vs. 4.5 ± 1.6 **	PIT is less cost-effective than ROS
**Pitavastatin vs. Atorvastatin**					
Thailand, 2008/2009 USD *** [29]	PIT 1 mg vs. ATOR 10 mg	-	% LDL-C reduction	0.77 * vs. 1.56 *	PIT is more cost-effective than ATOR
Korea, 2016 USD [31]	PIT 2 mg vs. ATOR 10 mg	183.1 vs. 216.0	% LDL-C reduction	8.0 ± 0.6 ** vs. 9.5 ± 0.5 **	PIT is more cost-effective than ATOR
India, 2020 RBI [30]	PIT 4 mg vs. ATOR 20 mg	-	% LDL-C reduction	43.68 vs. 40.41	PIT is less cost-effective than ATOR in T2DM patients within 12 weeks of treatment

Abbreviation: PIT: pitavastatin; ATOR: atorvastatin; ROS: rosuvastatin; LDL-C: low-density lipoprotein cholesterol; CKD: chronic kidney disease; variation is expressed as ±SD. *: monthly cost per % LDL-C reduction. **: yearly cost per % LDL-C reduction. ***: currency information was not explicitly stated; data were assumed based on the year the study took place.

**Table 4:** Data about incremental cost, QALY, and ICERs between pitavastatin and other statins.

Country, Currency	Comparison	Cost Types	Total Cost	QALY	ICERs (Cost/QALY)	Most Impactful Factor in DSA	Conclusion
Vietnam, 2024 USD [32]	PIT 2–4 mg vs. ATOR 10–20 mg PIT 2–4 mg vs. ROS 5–10 mg	Direct + drug	−1294 3693	−0.160 −0.072	8086 −51,404	Drug cost, utility	PIT is more cost-saving than ATOR but dominated by ROS at WTP = 14,155 USD/QALY

Abbreviation: PIT: pitavastatin; WTP: willingness to pay; QALY: quality-adjusted life year; ICER: incremental cost-effectiveness ratio.

## Data Availability

No new data were created or analyzed in this study.

## Supplementary Materials

The following supporting information can be downloaded at https://www.mdpi.com/article/10.3390/healthcare14131847/s1. Table S1. Multiple syntax attempts were made to retrieve research identification from the PubMed, Cochrane, and Embase databases; Table S2. Risk of bias assessment of RCT following RoB 2 checklist; Table S3. Risk of bias assessment of non-RCT following ROBIN I checklist.

## References

[1] Čereškevičius D., Zabiela V., Aldujeli A., Lesauskaitė V., Zubielienė K., Raškevičius V., Čiapienė I., Žaliaduonytė D., Giedraitienė A., Žvikas V. (2024). Impact of CYP2C19 Gene Variants on Long-Term Treatment with Atorvastatin in Patients with Acute Coronary Syndromes. Int. J. Mol. Sci..

[2] Reitzinger S., Reiss M., Czypionka T. (2024). Costs attributable to hypercholesterolemia in a single period and over the life cycle. Eur. J. Health Econ..

[3] Gyawali D., Vohra R., Orme-Johnson D., Ramaratnam S., Schneider R.H. (2021). A Systematic Review and Meta-Analysis of Ayurvedic Herbal Preparations for Hypercholesterolemia. Medicina.

[4] Alqahtani M.S., Alzibali K.F., Mahdi A.M.M., A Alharbi O.M., A Harbi R.H., Alkhaldi H.S.M., A Alsayafi Z.A., Albisher F.H., Buqurayn M.H., Alharbi M.M. (2024). Lipid-Lowering Medications for Managing Dyslipidemia: A Narrative Review. Cureus.

[5] Merćep I., Vujević A., Strikić D., Radman I., Pećin I., Reiner Ž. (2023). Present and Future of Dyslipidaemia Treatment—A Review. J. Clin. Med..

[6] Xia Q., Chen Y., Yu Z., Huang Z., Yang Y., Mao A., Qiu W. (2023). Prevalence, awareness, treatment, and control of dyslipidemia in Chinese adults: A systematic review and meta-analysis. Front. Cardiovasc. Med..

[7] Pirillo A., Norata G.D. (2023). The burden of hypercholesterolemia and ischemic heart disease in an ageing world. Pharmacol. Res..

[8] Li H., Zhao S., Wu J., Han J., Xu Y., Shi S., Zhang Y. (2024). Estimating the effect of inclisiran on hypercholesterolemia and primary prevention of cardiovascular disease: The NHANES 1999-2018 study. Lipids Health Dis..

[9] Choi W., Koo H., Jeong K.H., Kim E., You S.-H., Lee M.-T., Jung S.-Y. (2023). Therapeutic Duplication as a Medication Error Risk in Fixed-Dose Combination Drugs for Dyslipidemia: A Nationwide Study. Korean J. Clin. Pharm..

[10] Fox K.M., Wang L., Gandra S.R., Quek R.G.W., Li L., Baser O. (2016). Clinical and economic burden associated with cardiovascular events among patients with hyperlipidemia: A retrospective cohort study. BMC Cardiovasc. Disord..

[11] Kazi D.S., Elkind M.S., Deutsch A., Dowd W.N., Heidenreich P., Khavjou O., Mark D., Mussolino M.E., Ovbiagele B., Patel S.S. (2024). Forecasting the Economic Burden of Cardiovascular Disease and Stroke in the United States Through 2050: A Presidential Advisory From the American Heart Association. Circulation.

[12] Gasca-Pineda R., Osorio-Hernández M., Mehta R., Escobedo-De-La-Peña J., Narváez-Oriani C.A. (2023). Economic burden of hypercholesterolemia in high risk of cardiovascular disease population in Mexico. Arch. Cardiol. Mex..

[13] Boytsov S., Logunova N., Khomitskaya Y. (2017). Suboptimal control of lipid levels: Results from the non-interventional Centralized Pan-Russian Survey of the Undertreatment of Hypercholesterolemia II (CEPHEUS II). Cardiovasc. Diabetol..

[14] Shaw L.J., Goyal A., Mehta C., Xie J., Phillips L., Kelkar A., Knapper J., Berman D.S., Nasir K., Veledar E. (2018). 10-Year Resource Utilization and Costs for Cardiovascular Care. J. Am. Coll. Cardiol..

[15] Sciattella P., Maggioni A.P., Arcangeli E., Sidelnikov E., Kahangire D.A., Mennini F.S. (2022). Healthcare Resource Utilization, Cardiovascular Event Rate and Use of Lipid-Lowering Therapies in Secondary Prevention of ASCVD in Hospitalized Patients in Italy. Adv. Ther..

[16] Michaeli D.T., Michaeli J.C., Albers S., Boch T., Michaeli T. (2023). Established and Emerging Lipid-Lowering Drugs for Primary and Secondary Cardiovascular Prevention. Am. J. Cardiovasc. Drugs.

[17] Koren M.J., Rodriguez F., East C., Toth P.P., Watwe V., Abbas C.A., Sarwat S., Kleeman K., Kumar B., Ali Y. (2024). An “Inclisiran First” Strategy vs Usual Care in Patients With Atherosclerotic Cardiovascular Disease. J. Am. Coll. Cardiol..

[18] Martinho I., Rodrigues A.T., Guerreiro J., Rocha J., Sepodes B., Torre C. (2023). A cross-country utilization patterns comparison of high expenditure therapeutic groups between Portugal and six European countries: The two sides of the Portuguese coin. Expert Rev. Pharmacoeconomics Outcomes Res..

[19] Zhang Y., Zhang D., Liu X., Peng W., Mu Y., Li Y., Qiu Q. (2023). A Practical Statin Recommendation System Based on Real-World Data to Improve LDL-C Management in Secondary Prevention. J. Cardiovasc. Pharmacol..

[20] Mitani H., Suzuki K., Ako J., Iekushi K., Majewska R., Touzeni S., Yamashita S. (2023). Achievement Rates for Low-Density Lipoprotein Cholesterol Goals in Patients at High Risk of Atherosclerotic Cardiovascular Disease in a Real-World Setting in Japan. J. Atheroscler. Thromb..

[21] Lin F.-J., Shyu K.-G., Hsieh I.-C., Sheu W.H.-H., Tu S.-T., Yeh S.-J., Chen C.-I., Lu K.-C., Wu C.-C., Shau W.-Y. (2020). Cost-effectiveness of statin therapy for secondary prevention among patients with coronary artery disease and baseline LDL-C 70–100 mg/dL in Taiwan. J. Formos. Med. Assoc..

[22] Page M.J., McKenzie J.E., Bossuyt P.M., Boutron I., Hoffmann T.C., Mulrow C.D., Shamseer L., Tetzlaff J.M., Akl E.A., Brennan S.E. (2021). The PRISMA 2020 statement: An updated guideline for reporting systematic reviews. BMJ.

[23] Verhoef T.I., Morris S. (2015). Cost-effectiveness and pricing of antibacterial drugs. Chem. Biol. Drug Des..

[24] Campbell M., McKenzie J.E., Sowden A., Katikireddi S.V., Brennan S.E., Ellis S., Hartmann-Boyce J., Ryan R., Shepperd S., Thomas J. (2020). Synthesis without meta-analysis (SWiM) in systematic reviews: Reporting guideline. BMJ.

[25] Kunst N., Siu A., Drummond M., Grimm S.E., Grutters J., Husereau D., Koffijberg H., Rothery C., Wilson E.C., Heath A. (2023). Consolidated Health Economic Evaluation Reporting Standards-Value of Information (CHEERS-VOI): Explanation and Elaboration. Value Health.

[26] Sterne J.A.C., Savović J., Page M.J., Elbers R.G., Blencowe N.S., Boutron I., Cates C.J., Cheng H.Y., Corbett M.S., Eldridge S.M. (2019). RoB 2: A revised tool for assessing risk of bias in randomised trials. BMJ.

[27] The ROBINS-I Group (2024). Risk of Bias in Non-Randomized Studies-of Interventions, Version 2 (ROBINS-I V2). Archived Draft Version from November 2024. https://www.riskofbias.info/welcome/robins-i-v2.

[28] Abe M., Maruyama N., Maruyama T., Okada K., Soma M. (2015). A Trial of Pitavastatin Versus Rosuvastatin for Dyslipidemia in Chronic Kidney Disease. J. Atheroscler. Thromb..

[29] Sansanayudh N., Wongwiwatthananukit S., Putwai P., Dhumma-Upakorn R. (2010). Comparative efficacy and safety of low-dose pitavastatin versus atorvastatin in patients with hypercholesterolemia. Ann. Pharmacother..

[30] Devi G., Singh J., Bal B.P.S., Chaudhary S. (2025). Comparative Effectiveness of Pitavastatin Versus Atorvastatin on Lipid Profile and Blood Sugar in Patients of Diabetic Dyslipidemia: An Open-Label Comparative Study. Cureus.

[31] Jeong Y.J., Kim H., Baik S.J., Kim T.M., Yang S.J., Lee S.-H., Cho J.-H., Lee H., Yim H.W., Choi I.Y. (2017). Analysis and comparison of the cost-effectiveness of statins according to the baseline low-density lipoprotein cholesterol level in Korea. J. Clin. Pharm. Ther..

[32] Vo N.X., Nguyen H.T.M., Phan N.M., Pham H.L., Bui T.T., Bui T.T. (2025). Cost-Effectiveness Analysis of Pitavastatin in Dyslipidemia: Vietnam Case. Healthcare.

[33] Kohli-Lynch C.N., Bellows B.K., Thanassoulis G., Zhang Y., Pletcher M.J., Vittinghoff E., Pencina M.J., Kazi D., Sniderman A.D., Moran A.E. (2019). Cost-effectiveness of Low-density Lipoprotein Cholesterol Level-Guided Statin Treatment in Patients with Borderline Cardiovascular Risk. JAMA Cardiol..

[34] Wang M., Liu J., Bellows B.K., Qi Y., Sun J., Liu J., Moran A.E., Zhao D. (2020). Impact of China’s Low Centralized Medicine Procurement Prices on the Cost-Effectiveness of Statins for the Primary Prevention of Atherosclerotic Cardiovascular Disease. Glob. Heart.

[35] Zheng D., Ren J., Lv D., Zhao Q., Hong D. (2025). Ranking the Diabetes-related Safety Profile of Different Statin Drugs. Curr. Pharm. Des..

[36] Lin S.-Y., Baumann K., Zhou C., Zhou W., Cuellar A.E., Xue H. (2021). Trends in Use and Expenditures for Brand-name Statins After Introduction of Generic Statins in the US, 2002–2018. JAMA Netw. Open.

[37] Matyori A., Brown C.P., Ali A., Sherbeny F. (2023). Statins utilization trends and expenditures in the U.S. before and after the implementation of the 2013 ACC/AHA guidelines. Saudi Pharm. J..

[38] Li J., Gong J., Liu Z., Liu Y., He A., Wang Z. (2025). Safety Profile of Statins for Post-Marketing Adverse Cardiovascular Events: A Real-World Pharmacovigilance Analysis. Endocr. Metab. Immune Disord. Drug Targets.

[39] Zhou L., Wu B., Bian Y., Lu Y., Zou Y., Lin S., Li Q., Liu C. (2025). Hepatotoxicity associated with statins: A retrospective pharmacovigilance study based on the FAERS database. PLoS ONE.

[40] Ogura T., Shiraishi C. (2025). Comparative Analysis of Adverse Event Profiles Among Seven Statins for Hypercholesterolemia Management Using the United States FDA Adverse Event Reporting System. Cureus.

[41] FJ F.J., Ruiz E., Becerra V., Aceituno S., Ferrario M.G., Lizán L., Gracia A. (2015). Cost-effectiveness of rosuvastatin versus simvastatin, atorvastatin and pitavastatin in patients with high and very high cardiovascular risk in Spain. Clin. Investig. Arterioscler..

[42] Ikeda S., Kobayashi M. (2008). Pharmacoeconomic analysis of HMG-CoA reductase inhibitors (statins) in patients with hypercholesterolemia. Jpn. J. Healthc. Manag..

[43] Zhang X., Xing L., Jia X., Pang X., Xiang Q., Zhao X., Ma L., Liu Z., Hu K., Wang Z. (2020). Comparative Lipid-Lowering/Increasing Efficacy of 7 Statins in Patients with Dyslipidemia, Cardiovascular Diseases, or Diabetes Mellitus: Systematic Review and Network Meta-Analyses of 50 Randomized Controlled Trials. Cardiovasc. Ther..

[44] Li C., Spencer G., Husain M.J., Nugent R., Auzenne D., Kostova D., Richter P. (2024). Barriers to Accessibility of Medicines for Hyperlipidemia in Low- And Middle-Income Countries. PLoS Glob. Public Health.

[45] Husain M.J., Spencer G., Nugent R., Kostova D., Richter P. (2022). The Cost-Effectiveness of Hyperlipidemia Medication in Low- and Middle-Income Countries: A Review. Glob. Heart.

[46] Heller D.J., Coxson P.G., Penko J., Pletcher M.J., Goldman L., Odden M.C., Kazi D.S., Bibbins-Domingo K. (2017). Evaluating the Impact and Cost-Effectiveness of Statin Use Guidelines for Primary Prevention of Coronary Heart Disease and Stroke. Circulation.

[47] De Smedt D., Annemans L., De Backer G., Kotseva K., Rydèn L., Wood D., Amouyel P., Bruthans J., Cifkova R., De Sutter J. (2018). Cost-effectiveness of optimized adherence to prevention guidelines in European patients with coronary heart disease: Results from the EUROASPIRE IV survey. Int. J. Cardiol..

[48] Pichon-Riviere A., Drummond M., Palacios A., Garcia-Marti S., Augustovski F. (2023). Determining the efficiency path to universal health coverage: Cost-effectiveness thresholds for 174 countries based on growth in life expectancy and health expenditures. Lancet Glob. Health.

[49] Weise A., Büchter R.B., Pieper D., Mathes T. (2022). Assessing transferability in systematic reviews of health economic evaluations—A review of methodological guidance. BMC Med. Res. Methodol..

