# Supplementary material for: Cost-Effectiveness of Pitavastatin in Dyslipidemia: A Systematic Review
Source: Healthcare (Basel). 2026 Jun 25;14(13):1847. doi: 10.3390/healthcare14131847 (PMC13361831; doi:10.3390/healthcare14131847)
Supplement: Supplementary file 1 [file healthcare-14-01847-s001.zip › healthcare-4277955-supplementary.pdf]

healthcare

MDPI

Systematic Review

Cost-Effectiveness of Pitavastatin in Dyslipidemia: A Systematic Review

2.1. Searching strategy

Table S1. Multiple syntax attempts were made to retrieve research identification from the PubMed, Cochrane, and Embase databases.

No.	Syntax	Number of results
	**Pubmed**	
1	((cost-effectiveness) OR (cost-benefit)) AND (pitavastatin) AND ((dyslipidemia) OR (hypercholesterolemia))	6
2	((cost-effectiveness) OR (cost-benefit) OR (cost-effective)) AND (pitavastatin) AND ((dyslipidemia) OR (hypercholesterolemia))	6
3	(pitavastatin) AND (cost-effective)	7
4	((cost-effectiveness) OR (cost-effective) OR (economic evaluation) OR (cost-benefit) OR (cost-utility) OR (cost-minimization)) AND (pitavastatin) AND ((dyslipidemia) OR (hyperlipidemia) OR (hypercholesterolemia))	6
5	(pitavastatin) AND ((cost-effective) OR (cost-effectiveness) OR (economic evaluation)OR (cost-efficacy) OR (cost-utility) OR (cost-saving))	14
6	(pitavastatin) AND ((cost) OR (cost-effective) OR (cost-effectiveness))	20
	**Cochrane**	
1	((cost-effectiveness) OR (cost-effective) OR (economic evaluation) OR (cost-benefit) OR (cost-utility) OR (cost-minimization)) AND (pitavastatin) AND ((dyslipidemia) OR (hyperlipidemia) OR (hypercholesterolemia))	2
	**Embase**	
1	(((cost-effectiveness) OR (cost-effective) OR (economic evaluation) OR (cost-benefit) OR (cost-utility) OR (cost-minimization)) AND (pitavastatin) AND ((dyslipidemia) OR (hyperlipidemia) OR (hypercholesterolemia)))	53
2	(pitavastatin) AND ((cost-effective) OR (cost-effectiveness) OR (economic evaluation)OR (cost-efficacy) OR (cost-utility) OR (cost-saving))	84
3	(pitavastatin) AND ((cost) OR (cost-effective) OR (cost-effectiveness))	186

3.4. RoB 2 and ROBIN-I

Table S2. Risk bias assessment of RCT following RoB 2 checklist.

Study	D1	D2	D3	D4	D5	Overall
Abe et al., Japan 2015 [1]	Low	Low	Low	Low	Low	Low
Sansanayudh et al., Thailand 2010 [2]	Some concerns	Low	Low	Low	Low	Some concerns
Devi et al., India 2025 [3]	Some concerns	High	High	Low	Low	High

Table S3. Risk bias assessment of non-RCT following ROBIN I checklist.

Study	D1	D2	D3	D4	D5	D6	D7	Overall
Jeong et al., Korea 2017 [4]	Serious	Low	Serious	Serious	Serious	Low	Moderate	Serious

1. Abe, M.; Maruyama, N.; Maruyama, T.; Okada, K.; Soma, M. A Trial of Pitavastatin Versus Rosuvastatin for Dyslipidemia in Chronic Kidney Disease. *J Atheroscler Thromb* **2015**, *22*, 1235-47. doi: 10.5551/jat.29264
2. Sansanayudh, N.; Wongwiwatthananukit, S.; Putwai, P.; Dhumma-Upakorn, R. Comparative efficacy and safety of low-dose pitavastatin versus atorvastatin in patients with hypercholesterolemia. *Ann Pharmacother* **2010**, *44*, 415-23. doi: 10.1345/aph.1M522
3. Devi, G.; Singh, J.; Bal, B.P.S.; Chaudhary, S. Comparative Effectiveness of Pitavastatin Versus Atorvastatin on Lipid Profile and Blood Sugar in Patients of Diabetic Dyslipidemia: An Open-Label Comparative Study. *Cureus* **2025**, *17*, e90307. doi: 10.7759/cureus.90307
4. Jeong, Y.J.; Kim, H.; Baik, S.J.; Kim, T.M.; Yang, S.J.; et al. Analysis and comparison of the cost-effectiveness of statins according to the baseline low-density lipoprotein cholesterol level in Korea. *J Clin Pharm Ther* **2017**, *42*, 292-300. doi: 10.1111/jcpt.12512
